# Interventions and Outcomes for Neoadjuvant Treatment of T4 Colon Cancer: A Scoping Review

**DOI:** 10.3390/curroncol28030191

**Published:** 2021-05-29

**Authors:** Flora Jung, Keegan Guidolin, Michael Ho-Yan Lee, Kimberley Lam-Tin-Cheung, Grace Zhao, Sachin Doshi, Tyler Chesney, Marina Englesakis, Jelena Lukovic, Grainne O’Kane, Fayez A. Quereshy, Sami A. Chadi

**Affiliations:** 1 Faculty of Medicine, University of Toronto, Toronto, ON M5S 1A8, Canada; flora.jung@mail.utoronto.ca (F.J.); michaelh.lee@mail.utoronto.ca (M.H.-Y.L.); grace.zhao@mail.utoronto.ca (G.Z.); 2 Department of Surgery, University of Toronto, Toronto, ON M5T1P5, Canada; keegan.guidolin@gmail.com (K.G.); kimberley.lamtincheung@mail.utoronto.ca (K.L.-T.-C.); sachin.doshi@mail.utoronto.ca (S.D.); tyler.chesney@unityhealth.to (T.C.); fayez.quereshy@uhn.ca (F.A.Q.); 3 Department of Surgery, St. Michael’s Hospital, Toronto, ON M5B 1W8, Canada; 4 Library and Information Services, University Health Network, Toronto, ON M5G 2C4, Canada; marina.englesakis@uhn.ca; 5 Radiation Medicine Program, Princess Margaret Cancer Centre, Toronto, ON M5G 2C1, Canada; Jelena.Lukovic@rmp.uhn.ca; 6 Department of Radiation Oncology, University of Toronto, Toronto, ON M5T 1P5, Canada; 7 Princess Margaret Cancer Centre, Division of Medical Oncology, Toronto, ON M5G 2C1, Canada; 8 Princess Margaret Cancer Centre, Division of Surgical Oncology, University Health Network, Toronto, ON M5G 2C1, Canada

**Keywords:** locally advanced colon cancer, T4 colon cancer, neoadjuvant therapy, chemotherapy, radiotherapy, chemoradiotherapy

## Abstract

While adjuvant treatment of colon cancers that penetrate the serosa (T4) have been well-established, neoadjuvant strategies have yet to be formally evaluated. Our objective was to perform a scoping review of eligibility criteria, treatment regimens, and primary outcomes for neoadjuvant approaches to T4 colon cancer. A librarian-led, systematic search of MEDLINE, Embase, Cochrane Library, Web of Science, and CINAHL up to 11 February 2020 was performed. Primary research evaluating neoadjuvant treatment in T4 colon cancer were included. Screening and data abstraction were performed in duplicate; analyses were descriptive or thematic. A total of twenty studies were included, most of which were single-arm, single-center, and retrospective. The primary objectives of the literature to date has been to evaluate treatment feasibility, tumor response, disease-free survival, and overall survival in healthy patients. Conventional XELOX and FOLFOX chemotherapy were the most commonly administered interventions. Rationale for selecting a specific regimen and for treatment eligibility criteria were poorly documented across studies. The current literature on neoadjuvant strategies for T4 colon cancer is overrepresented by single-center, retrospective studies that evaluate treatment feasibility and efficacy in healthy patients. Future studies should prioritize evaluating clear selection criteria and rationale for specific neoadjuvant strategies. Validation of outcomes in multi-center, randomized trials for XELOX and FOLFOX have the most to contribute to the growing evidence for this poorly managed disease.

## 1. Introduction

While 10 to 15% of patients with colon cancer are diagnosed with disease that penetrates the colonic serosa (T4), the outcomes for T4 colon cancer remain poor [1,2]. Multivisceral resection is often required to achieve negative margins in addition to adjuvant chemo- or radio-therapy for curative intent [3,4,5,6]. The three-year recurrence for patients with T4 colon cancer remains at 47% compared to 13% for those with T1-2 disease [7]. 

Neoadjuvant therapy is one strategy which has shown promise in improving oncologic outcomes for T4 colon cancer. It has demonstrated efficacy in gastric and rectal cancers and is postulated to contribute to the eradication of micrometastases and the reduction of cell shedding during resection [8,9,10,11,12,13,14]. The 2012 FOxTROT (fluoropyrimidine, oxaliplatin, and targeted receptor pre-operative therapy) study is the only randomized, controlled trial to date to evaluate neoadjuvant approaches to T4 colon cancer [15]. It compared neoadjuvant FOLFOX (folinic acid, 5-fluorouracil, and oxaliplatin) chemotherapy to the conventional direct-to-surgery approach in 150 patients and reported significant improvement in negative margin resection with no negative effects on postoperative morbidity [15]. Based largely on this data, the National Comprehensive Cancer Network added neoadjuvant chemotherapy as a treatment option for T4b disease in 2016; however, long-term outcome data supporting this recommendation are sparse [16]. Results from phase III of the FOxTROT trial, which evaluated 1053 patients with T3-4 colon cancer, are limited; however, their preliminary results presented in 2019 are encouraging [17]. Neoadjuvant FOLFOX is reportedly associated with tumor downstaging, pathologic complete response, near-complete tumor regression, negative margin resection, and reduced perioperative morbidity. 

Given the range of possible management approaches and characteristics of T4 tumors, a detailed summary of the current literature is required to identify and advance effective strategies. Our primary objectives in this scoping review were to describe outcomes of interest, regimens, rationale, and eligibility of current neoadjuvant strategies—including chemotherapy, radiotherapy, and chemoradiotherapy—for T4 colon cancer.

## 2. Materials and Methods

Our review protocol was developed a priori and submitted to Open Science Framework (OSF) on 16 August 2021 (https://osf.io/q7cjd) using methods recommended by the Joanna Briggs Institute (JBI) [18] and the PRISMA-ScR extension [19]. 

### 2.1. Systematic Search of the Literature

We searched five electronic databases up to 11 February 2020: MEDLINE (OVID); EMBASE (OVID); the Cochrane Central Register of Controlled Trials (CENTRAL; via OVID); Web of Science (Clarivate); and the Cumulative Index to Nursing & Allied Health Literature (CINAHL; via EbscoHost). Our search strategy was developed and executed by a medical librarian with a specialization in general surgery research (M.E.). MeSH (Medical subject headings) and keywords were identified through an initial limited search of MEDLINE and agreed upon by the research team. The final search strategy for MEDLINE via Ovid (available in full in Table S1) was translated by M.E. to meet requirements of the remaining databases. To maximize the number of eligible studies, no restrictions for language or publication status were used. Reference lists of included studies were screened to identify any other texts of relevance. 

### 2.2. Eligibility Criteria and Outcomes

We considered all primary research studies evaluating neoadjuvant treatment of T4 colon cancer. Studies which exclusively evaluated early stage (T1/T2/T3), metastatic, or recurrent colon cancers were excluded; studies evaluating neoadjuvant treatment of both T4 and early stage cancers were included. Studies that did not primarily aim to evaluate neoadjuvant treatment were excluded. There were no relevant exclusion criteria based on participant characteristics, including age, sex, race, culture, or comorbidity. Similarly, there were no relevant exclusion criteria based on study context, including by geographic location or healthcare setting. Our primary outcomes were treatment-selection criteria, treatment characteristics, and primary outcomes reported by each study.

### 2.3. Article Selection

After pilot-testing the eligibility criteria, all records were uploaded into Covidence software (www.covidence.org, accessed 10 April 2020) for the article selection process. Level 1 screening of all titles and abstracts was conducted to exclude clearly irrelevant citations. All citations were reviewed by F.J. and one other independent reviewer (M.L., S.D., G.Z., K.C.). Conflicts at this stage were resolved through automatic retention to ensure no relevant article was missed. Level 2 screening of full texts was performed similarly in duplicate for final inclusion of articles. Discrepancies at this stage were resolved by consensus; a third reviewer (K.G.) was consulted for any persisting disagreements. Reviewers were not blinded to author or journal name. 

### 2.4. Data Extraction and Analysis

A data abstraction form was developed *a priori* and pilot tested. Extracted information included study characteristics (year of publication, country of origin, study design, sample size, sample source, data source, inclusion and exclusion criteria, study limitations, recommended next steps), intervention details (intervention arms, evaluated therapies, dosing and delivery specifics), and measured outcomes (primary outcomes, reported effects). A comments section was used to capture any remaining relevant information not captured by the existing a priori fields. Studies were charted in duplicate using a narrative approach, with discordance resolved through consensus. Missing data were treated as not reported. Descriptive numerical analyses through frequency analysis were performed according to study designs, sample specifics, and types of interventions. Thematic analyses were performed where appropriate to evaluate qualitative data. 

## 3. Results

### 3.1. Literature Search and Selection Process

**Figure 1:**
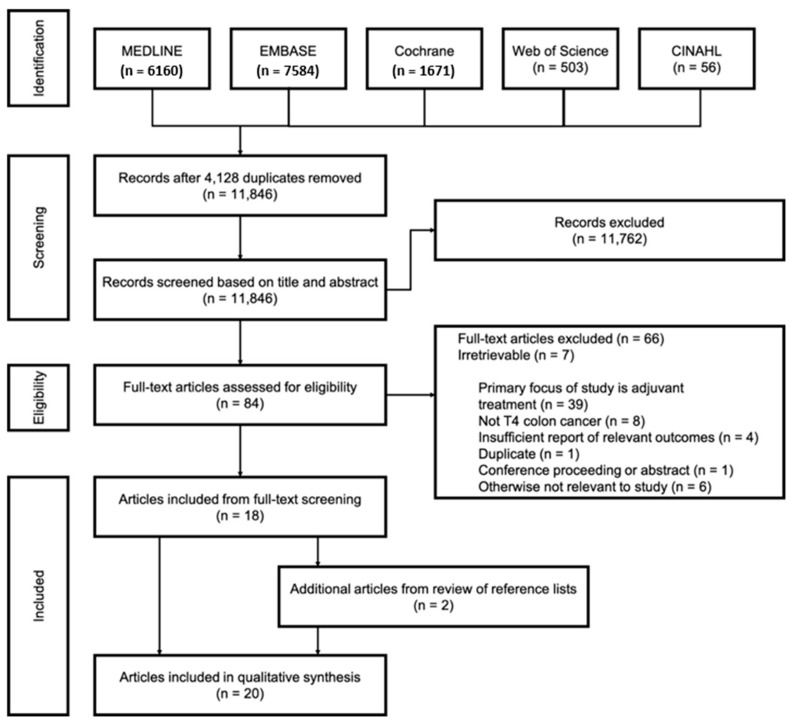
Article selection.

Our search identified 11,846 unique records after 4128 duplicates were removed (Figure 1). After duplicated abstract screening, 11,762 titles were identified as irrelevant, and the remaining 84 full-text articles were appraised for eligibility. Of these, 18 studies were identified from full-text screening and an additional 2 studies from reference lists; ultimately, 20 articles were included in our scoping review [15,20,21,22,23,24,25,26,27,28,29,30,31,32,33,34,35,36,37]. A very small number of studies, 7 of 11,846 articles (0.06%), were irretrievable using multi-institutional search of printed material through an inter-library database and multiple requests sent to corresponding authors.

### 3.2. Study Characteristics

Characteristics of the included studies are detailed in Table 1. We included 13 retrospective cohort studies, 6 prospective cohort studies, and 1 randomized control trial. The majority of these (n = 15) were published between 2015 and 2020. Study sample sizes ranged from 4 to 27,575 (median (IQR) 111.5 (33.75–145.25)). Most studies were single-center trials (n = 14) with data linkage to institutional health administrative data (n = 12). Three studies originating from USA linked to the same registry (National Cancer Database); although the study periods for these overlapped, each evaluated a different neoadjuvant therapy [25,27,29]. 

### 3.3. Participant Eligibility Criteria Used in Current Literature

Participant eligibility criteria used across studies are summarized in Table S2. Conditions for inclusion were thematically categorized into eight domains, of which three were patient-specific and five were disease-specific. All studies explicitly included patients with T4 colon cancer. The next most commonly reported criteria were age ≥18 and absence of significant co-morbidity as defined by the authors. 

Conditions for exclusion were similarly thematically categorized into eight domains, of which four were patient-specific, two were disease-specific, and three were intervention-specific. The most commonly reported criteria for exclusion were the presence of significant co-morbidity as defined by the authors, history of malignant disease, and age > 75. 

### 3.4. Outcomes Assessed in Studies of Neoadjuvant Therapy

**Figure 2:**
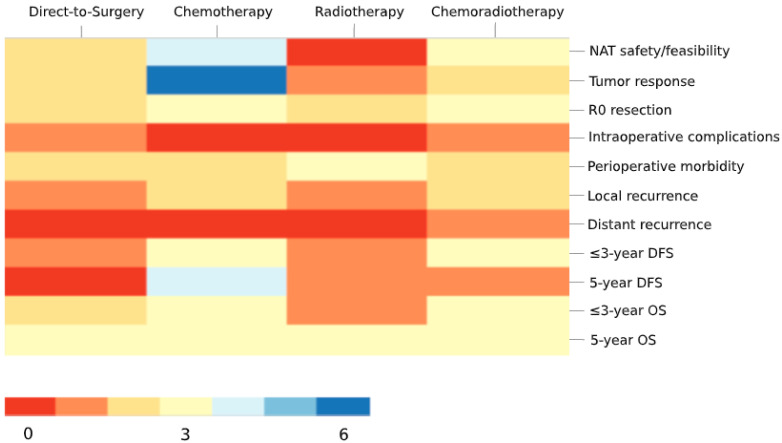
Heatmap graphic showing the distribution of primary outcomes in the current literature on neoadjuvant therapy for T4 colon cancer. Each box in the grid represents the number of studies (color coded per the legend) that use the primary outcome listed on the *y*-axis to evaluate the neoadjuvant therapy listed on the *x*-axis. NAT, neoadjuvant therapy; R0, microscopically negative margins; DFS, disease-free survival; OS, overall survival.

Primary outcomes used to evaluate neoadjuvant therapy are summarized in Figure 2, thematically categorized into 11 domains. Both short- and long-term postoperative outcomes of neoadjuvant therapy have been evaluated across studies with up to five years of follow-up data. The most commonly reported objective in the current neoadjuvant therapy literature is to evaluate neoadjuvant therapy safety and feasibility in addition to tumor-level responses. Cancer recurrence has been evaluated as a categorical outcome and as a time-to-event endpoint up to five years. While evaluation of local recurrence, three- and five-year disease-free survival was not uncommon, distant recurrence has only been reviewed by one study. 

### 3.5. Neoadjuvant Treatment Regimens for T4 Colon Cancer

**Figure 3:**
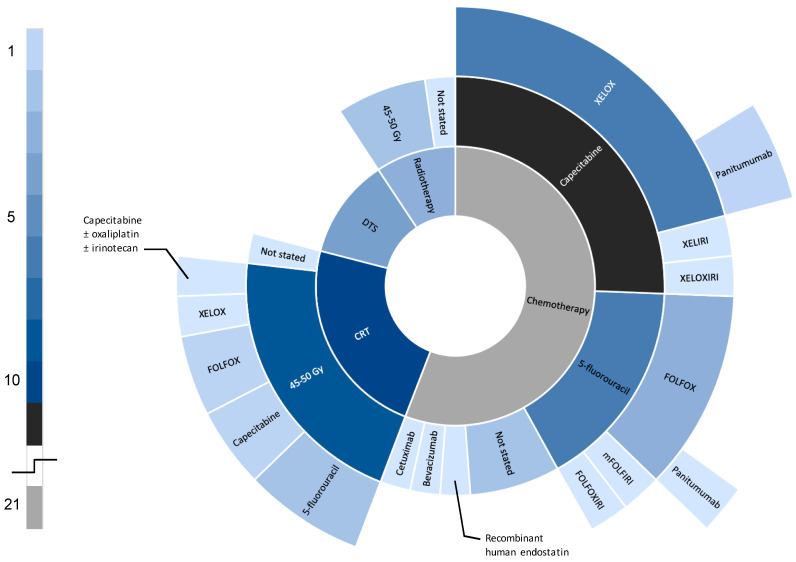
Neoadjuvant therapies in the current literature. (Categories are not mutually exclusive. Some trials evaluated more than one neoadjuvant therapy). DTS, direct-to-surgery; CRT, chemoradiotherapy; FOLFOX, folinic acid + 5-fluorouracil + oxaliplatin; FOLFOXIRI, FOLFOX + irinotecan; mFOLFIRI: 5-fluorouracil + irinotecan; XELOX, capecitabine + oxaliplatin; XELOXIRI, XELOX + irinotecan; XELIRI, capecitabine + irinotecan; NAC, neoadjuvant chemotherapy; NAR, neoadjuvant radiotherapy.

Chemotherapy was the most commonly evaluated neoadjuvant therapy to date (n = 11), followed by chemoradiotherapy (n = 8) and radiotherapy (n = 4) (Figure 3). Specific neoadjuvant regimens reported by included studies have been documented in Table 2. We found the most evidence for XELOX (capecitabine + oxaliplatin; n = 5) administered as oral capecitabine 1000 mg/m^2^ twice daily on days 1–14 + IV oxaliplatin 130 mg/m^2^ on day 1. The FOLFOX regimen used by the FOxTROT randomized control trial was reported as follows: oxaliplatin 85 mg/m^2^ + l-folinic acid 175 mg/m^2^ + fluorouracil 400 mg/m^2^; those patients with Kras wild-type cancers were sub-randomized with ± panitumumab 6mg/kg by IV bolus and 2400 mg/m^2^ by 46-h infusion. XELOX, FOLFOX, and panitumumab were the only chemotherapies that were evaluated by more than one study group. The remaining seven neoadjuvant chemotherapies had only single-study data. 

Three studies described the role of neoadjuvant radiation therapy, which was delivered either alone or with concurrent chemotherapy. The most commonly used dose/fractionation schedules were: 45 Gy in 25 fractions, 50.4 Gy in 28 fractions, and a simultaneous boost technique delivering 46 Gy/50 Gy in 25 fractions. Radiation therapy was delivered using either 3D-CRT (3-dimensional conformal radiotherapy) or IMRT (intensity-modulated radiotherapy). CT (computerized tomography) and/or MRI (magnetic resonance imaging) was used for simulation. The primary gross tumor volume (GTV) was defined as the visible tumor and any enlarged lymph nodes on imaging and identification of gross tumor volume (GTV) [28,33,35]. For patients with locally advanced colon cancer, the clinical target volume (CTV) was defined the GTV plus a 15 to 20 mm margin with an additional 10 to 15 mm margin for the PTV to account for organ motion. No elective nodal irradiation was done. In the setting of locally advanced sigmoid colon cancer, the CTV included the GTV plus a craniocaudal expansion of 2–3 cm and adjacent lymphatic drainage regions up to L4/L5. A 6 mm PTV margin was used. Dose constraints were described by all three studies [28,33,35]. 

### 3.6. Rationale and Treatment Eligibility for Neoadjuvant Therapy

**Figure 4:**
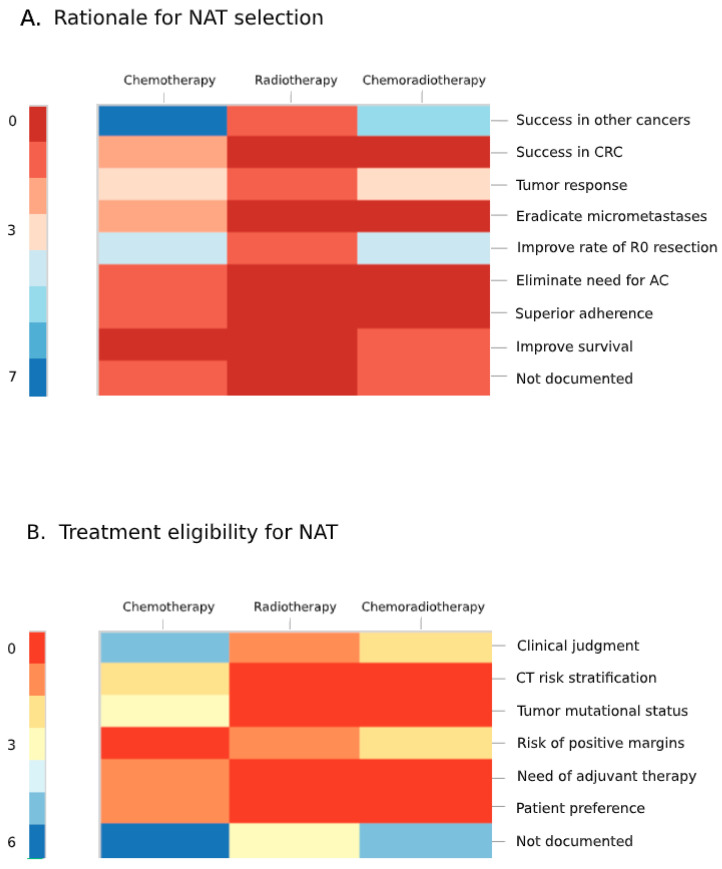
Heatmap graphics showing the rationale (**A**) and eligibility criteria (**B**) for neoadjuvant therapy for T4 colon cancer. Each box in the grid represents the number of studies (color coded per the legend) that use the rationale (**A**) or eligibility criteria (**B**) listed on the *y*-axis, corresponding to the neoadjuvant therapy listed on the *x*-axis. (Categories are not mutually exclusive). NAT, neoadjuvant therapy; CRC, colorectal cancer; R0, microscopically negative margins; AC, adjuvant chemotherapy; CT, computed tomography.

Rationale and treatment eligibility criteria for neoadjuvant therapy are summarized in Figure 4. Rationale was largely based on treatment category (i.e., chemotherapy, radiotherapy, and chemoradiotherapy), and rationale for specific regimens (e.g., FOLFOX or 45 Gy of radiation) were only defined by two studies (10%). Chemotherapy was most commonly selected for its success in achieving negative margin resection and micrometastases eradication in other cancers. Radiotherapy and chemoradiotherapy were selected for their success in achieving negative margin resection and tumor response in other cancers. Two studies identified a rationale for a specific regimen; one selected FOLFOX for its success in achieving pathologic complete response in rectal cancers [35], while the other selected FOLFOXIRI (FOLFOX + irinotecan) to test the benefit of combining three active chemotherapeutics [38]. 

Treatment eligibility criteria were also poorly documented across studies (n = 14) and otherwise largely based on clinical judgment. Chemotherapeutics had the most objective criteria, with some studies utilizing CT-based risk stratification and/or tumor mutational status. 

### 3.7. Summary of Findings for Neoadjuvant Treatment for T4 Colon Cancer

Across the 20 included studies, the most commonly reported conclusion was that neoadjuvant therapy is feasible (n = 12) and effective (n = 16) (Table S3). The most commonly reported study limitations were a retrospective design (n = 11) and small sample size (n = 9). The most commonly recommended next steps were validation of outcomes in a randomized and/or prospective trial (n = 12).

## 4. Discussion

Our scoping review identified 20 studies on neoadjuvant treatments for T4 colon cancer. These studies largely excluded extremes of age (less than 18 years and over 75 years), significant co-morbidity, and history of other malignancy. Evaluation of safety, feasibility, and postoperative outcomes up to five years were the primary focus, particularly for XELOX and FOLFOX ± panitumumab. However, treatment eligibility and administration were poorly documented, or inconsistent within samples.

Strategies using neoadjuvant XELOX and FOLFOX for T4 colon cancers derive from established success in metastatic colon cancers, which have established tumor response rates exceeding 50% [39,40]. This is significant in the setting of T4 colon cancers, as preoperative tumor downsizing may reduce incomplete resection and local recurrence rates [7,8,41,42]. More recent strategies have incorporated EGFR (epidermal growth factor receptor)-targeted monoclonal antibodies, panitumumab and cetuximab. These achieve even higher tumor responses in metastatic colon cancers when administered with an oxaliplatin-based chemotherapy for Kras wild-type tumors; however, those with Kras mutations experience poor effectiveness, and mandated preoperative testing was introduced for treatment eligibility [43,44,45,46,47,48,49]. We identified four studies which have evaluated panitumumab and cetuximab in T4 colon cancer [15,20,34,36]. Only one of these four listed known colon cancer mutational status as a criterion for study inclusion, though all reported ascertainment of mutational status prior to administration of these antibodies [20]. The role of neoadjuvant panitumumab and cetuximab in T4 colon cancers remains unclear. No studies compared different chemotherapy strategies head-to-head.

A retrospective design was the most commonly reported study limitation in the current literature (n = 11, 55%). Authors acknowledged that the administration of neoadjuvant regimens within their selected cohort was non-standardized and subsequently lead to inconsistencies without explicit clinical reasoning [26,33,36,37]. Furthermore, almost half of the studies failed to report specifics for the treatment regimen they evaluated, such as dose and dosing interval. While the ability to regulate patient selection and interventions may be innately more challenging in retrospective design studies, the overrepresentation of such data in the current literature is concerning. To advance this literature, administration of specific neoadjuvant regimens must be standardized, and any regimens tested by future studies should be described at sufficient detail which facilitates duplication for further evaluation. 

It should be highlighted that there is also concern in the current literature for bias and poorly-documented rationale in patient identification for neoadjuvant treatment; one double-arm, retrospective study of neoadjuvant radiotherapy in 131 patients acknowledged that treatment eligibility was based on an oncologist or surgeon’s judgment over explicit clinical or radiographic criteria [26]. The majority of other studies included in our review provided no rationale at all. Advancements in CT have permitted accurate identification of high-risk T4 tumors [7,15,50,51]. There has been recognition that FOLFOX and XELOX perform similarly in adjuvant data, though the same has not been replicated in neoadjuvant settings [52]. Heterogeneity in outcomes may thus be more apparent in the context of comparing chemotherapy with concurrent radiation and/or antibody use. A CT risk stratification algorithm has been proposed, through a retrospective study of 121 patients undergoing primary resection for non-metastatic colon cancer, as a robust method for identifying those who may benefit from neoadjuvant treatment based on extramural depth of the tumor; adoption of such criteria may reduce subjectivity in patient identification for neoadjuvant treatment [7]. Further evaluation of explicit selection criteria for different neoadjuvant regimens and the oncologic outcomes in patients who meet them are recommended for future studies. In addition, inclusion of more diverse outcomes, such as quality of life, should be included in future studies, as these remain poorly studied in the existing literature.

The most commonly reported next step to address existing limitations across studies was validation of outcomes in randomized or prospective trials. The aforementioned multi-center, phase III FOxTROT trial was one seeking to evaluate pathologic downstaging and recurrence in response to FOLFOX ± panitumumab; recruitment of patients with T3-4 colon cancer for this study was completed in December 2016 [15,16,17]. Another is the phase II Prodige 22-Eckinoxe trial seeking to evaluate tumor regression in response to FOLFOX ± cetuximab in patients similarly with T3-4 colon cancer; estimated completion date of recruitment for this study remains February 2021 [53]. Results from these and other planned, randomized trials of neoadjuvant therapies are awaited to further the current level of evidence; to corroborate existing data from single-arm and retrospective studies, evaluation of XELOX or radiotherapy in a randomized trial may also be beneficial. 

The conclusions of our review have limitations. Our team decided by consensus to exclude studies which had a primary aim of evaluating adjuvant treatment in patients with T4 colon cancer, although these studies may also include those who received prior neoadjuvant treatment. This decision was based on pilot testing of our eligibility criteria, which found a lack of patients and outcomes of interest reported by such articles. We mitigated the potential impact of this decision through performance of extensive full-text reviews with four independent members of our team and are reassured that this did not significantly affect our collection of relevant outcomes in the literature. Secondly, scoping reviews are based on categorization of articles and thematic classification of schemes by nature and thus are subject to the innate limitations of descriptive analyses. We mitigated potential for subjectivity through a predefined, pilot-tested data extraction sheet and duplicated abstractions. 

## 5. Conclusions

Literature to date on neoadjuvant strategies for T4 colon cancer has evaluated feasibility, tumor response, and survival in healthy patients. Clear selection criteria for different neoadjuvant strategies in T4 colon cancer have not been defined or evaluated, and we strongly recommend future studies to prioritize this in order to optimize treatment for patients who may benefit and avoid overtreatment of those who would not. Large, multi-center, randomized trials which further assess this for FOLFOX and XELOX will have the most to contribute. 

## Figures and Tables

**Table 1:** Selected study characteristics of included articles (n = 20).

Study Characteristic	No. (%)
Year published	
2011–2014	5 (25)
2015–2019	15 (75)
Country of origin	
USA	6 (30)
Canada	1 (5)
UK	1 (5)
Denmark	2 (10)
Spain	2 (10)
China	6 (30)
Taiwan	1 (5)
Japan	1 (5)
Design	
Randomized control trial	1 (5)
Retrospective cohort	13 (65)
Prospective cohort	6 (30)
Comparative arm	
Yes	6 (30)
No	14 (70)
Sample source	
Single-center	14 (70)
Multi-center	4 (20)
Not stated	2 (10)
Data source	
Institutional data	12 (60)
National Cancer Database	3 (15)
Cleveland Clinic colorectal cancer registry	1 (5)
Colorectal Carcinoma Database	1 (5)
Not stated	3 (15)

**Table 2:** Neoadjuvant regimens as detailed by included studies.

Treatment Regimen [ref]	Duration (Weeks)	Dose Interval (Weeks)
Direct-to-Surgery [15,25,26,27,28]		
Chemotherapy		
FOLFOX Oxaliplatin 85 mg/m^2^ + l-folinic acid 175 mg/m^2^ + fluorouracil 400 mg/m^2^, then ± panitumumab 6 mg/kg by IV bolus and 2400 mg/m^2^ by 46 h infusion [15]	6	2
No dosing details described [37]		
FOLFOXIRI 1 h infusion of irinotecan 150 mg/m^2^ on day 1, then 120 min infusion of oxaliplatin 85 mg/m^2^ + l-folinic acid 200 mg/m^2^ on day 2, then 5-fluorouracil 500 mg IV bolus and 2400 mg/m^2^ by 44 h infusion or S-1 40–60 mg orally BID for 10 days or capecitabine 1000 mg/m^2^ orally BID for 10 days [23]	-	2
mFOLFIRI [36]		
XELOX Capecitabine 1000 mg/m^2^ orally BID on days 1–14 + oxaliplatin 130 mg/m^2^ by IV on day 1 ± panitumumab 9 mg/kg IV [20,24,34]	9	3
No dosing details described [20,21,24,34,36]		
XELOXIRI, no dosing details described [36]		
XELIRI, no dosing details described [36]		
Cetuximab, no dosing details described [36]		
Bevacizumab, no dosing details described [36]		
Recombinant human endostatin, no dosing details described [36]		
Not specified [25,30,38]		
Radiation		
External beam radiation, 45–50 Gy/25 fractions [26,27,31]	5	-
Not specified [38]		
Chemoradiation		
External beam radiation, 45–50 Gy/25 fractions with		
FOLFOX Oxaliplatin 85 mg/m^2^ + l-folinic acid 400 mg/m^2^ by IV bolus, then 46 h infusion of 5-fluorouracil 2800 mg/m^2^ [35]	-	2
Oxaliplatin 80 mg/m^2^ + l-folinic acid 300 mg/m^2^/d on day 1–5 + 5-fulorouracil 400 mg/m^2^/d on day 1–4 [28]	3	-
No dosing details described [26]		
5-FU 5-fluorouracil 225 mg/m^2^/day + 45–50 Gy, 25 fractions [32] No dosing details described [38]	-	-
XELOX Capecitabine 1000 mg/m^2^ orally BID on days 1–14 ± oxaliplatin 100 mg/m^2^ on day 1 [22]	-	-
Capecitabine only, no dosing details described [22]		
mFOLFOX or FOLFOXIRI or XELOX, no dosing details described [33]	10	5
Not specified [29]		

Categories are not mutually exclusive. Some trials evaluated more than one neoadjuvant therapy; -: not reported; FOLFOX, folinic acid + 5-fluorouracil + oxaliplatin; FOLFOXIRI, FOLFOX + irinotecan; mFOLFIRI, 5-fluorouracil + irinotecan; XELOX, capecitabine + oxaliplatin; XELOXIRI, XELROX + irinotecan; XELIRI, capecitabine + irinotecan; NAC, neoadjuvant chemotherapy; NAR, neoadjuvant radiotherapy.

## Data Availability

Not applicable.

## Supplementary Materials

The following are available online at https://www.mdpi.com/article/10.3390/curroncol28030191/s1, Table S1: Full search strategy for Ovid MEDLINE^®^ 1946 to 10 February 2020; Table S2: Distribution of inclusion and exclusion criteria for study participant eligibility; Table S3: Summary of conclusions from included studies.
