# Supplementary material for: Interventions and Outcomes for Neoadjuvant Treatment of T4 Colon Cancer: A Scoping Review
Source: Curr Oncol. 2021 May 29;28(3):2065–78. doi: 10.3390/curroncol28030191 (PMC8261638; doi:10.3390/curroncol28030191)
Supplement: Supplementary file 1 [file curroncol-28-00191-s001.zip › curroncol-1198574-supplementary.pdf]

Current Oncology

MDPI

Supplementary Materials

Interventions and Outcomes for Neoadjuvant Treatment of T4 Colon Cancer: A Scoping Review

Flora Jung, Keegan Guidolin, Michael Ho-Yan Lee, Kimberley Lam Tin Cheung, Grace Zhao, Sachin Doshi, Tyler Chesney, Marina Englesakis, Jelena Lukovic, Grainne O'Kane, Fayez A. Quereshy, and Sami A. Chadi

Table S1. Full search strategy for Ovid MEDLINE(R) 1946 to February 10, 2020. *[Online only].*

#	Searches	Results
1	exp Colorectal Neoplasms/	196691
2	(adenocarcinom* adj3 (colorect* or colon* or rect* or intestine* or large bowel* or bowel* or anal or anus or perianal or peri-anal or circumanal or sigmoid*)).mp.	15427
3	(adenom* adj3 (colorect* or colon* or rect* or intestine* or large bowel* or bowel* or anal or anus or perianal or peri-anal or circumanal or sigmoid*)).mp.	8289
4	(cancer* adj3 (colorect* or colon* or rect* or intestine* or large bowel* or bowel* or anal or anus or perianal or peri-anal or circumanal or sigmoid*)).mp.	146926
5	(carcinom* adj3 (colorect* or colon* or rect* or intestine* or large bowel* or bowel* or anal or anus or perianal or peri-anal or circumanal or sigmoid*)).mp.	39489
6	(malignan* adj3 (colorect* or colon* or rect* or intestine* or large bowel* or bowel* or anal or anus or perianal or peri-anal or circumanal or sigmoid*)).mp.	5645
7	(metasta* adj3 (colorect* or colon* or rect* or intestine* or large bowel* or bowel* or anal or anus or perianal or peri-anal or circumanal or sigmoid*)).mp.	24040
8	(neoplas* adj3 (colorect* or colon* or rect* or intestine* or large bowel* or bowel* or anal or anus or perianal or peri-anal or circumanal or sigmoid*)).mp.	194876
9	(tumor* adj3 (colorect* or colon* or rect* or intestine* or large bowel* or bowel* or anal or anus or perianal or peri-anal or circumanal or sigmoid*)).mp.	23428
10	(tumour* adj3 (colorect* or colon* or rect* or intestine* or large bowel* or bowel* or anal or anus or perianal or peri-anal or circumanal or sigmoid*)).mp.	4854
11	(Sessile adj4 polyp*).mp.	997
12	(pedunculated adj4 polyp*).mp.	568
13	(colon?? adj2 polyp*).mp.	10393
14	or/1-13 [Colon/Colorectal Cancer & related terms]	250155
15	exp Surgical Procedures, Operative/ and (exp Abdomen/ or exp Digestive System Diseases/ or Digestive System/)	450568
16	su.fs. and (exp Abdomen/ or exp Digestive System Diseases/ or Digestive System/)	368322
17	Surgeons/ and (exp Abdomen/ or exp Digestive System Diseases/ or Digestive System/)	476
18	exp Intraoperative Care/ and (exp Abdomen/ or exp Digestive System Diseases/ or Digestive System/)	2978
19	exp Intraoperative Complications/ and (exp Abdomen/ or exp Digestive System Diseases/ or Digestive System/)	7099
20	exp Intraoperative Period/ and (exp Abdomen/ or exp Digestive System Diseases/ or Digestive System/)	6458
21	exp Postoperative Care/ and (exp Abdomen/ or exp Digestive System Diseases/ or Digestive System/)	8901
22	exp Postoperative Period/ and (exp Abdomen/ or exp Digestive System Diseases/ or Digestive System/)	7524

23	exp Postoperative Complications/ and (exp Abdomen/ or exp Digestive System Diseases/ or Digestive System/)	95520
24	adrenalectom*.mp.	24658
25	appendectom*.mp.	13372
26	arthrectom*.mp.	32
27	cholecystectom*.mp.	34861
28	colectom*.mp.	21756
29	cystectom*.mp.	14616
30	duodenectom*.mp.	612
31	fundectom*.mp.	87
32	gastrectom*.mp.	41251
33	glossectom*.mp.	1283
34	hemicolectom*.mp.	3288
35	hemi-colectom*.mp.	82
36	hepatectom*.mp.	34692
37	hypophysectom*.mp.	14253
38	jejunectom*.mp.	84
39	laparoscop*.mp.	120488
40	laparotom*.mp.	52376
41	lymphadenectom*.mp.	15170
42	lymph-adenectom*.mp.	61
43	lymphectom*.mp.	46
44	mesohepatectom*.mp.	57
45	meso-hepatectom*.mp.	1
46	metastasectom*.mp.	2144
47	necrosectom*.mp.	798
48	(operable or operabilit*).mp. and (exp Abdomen/ or exp Digestive System Diseases/ or Digestive System/)	1760
49	((operable or operabilit*) and (abdomen or abdominal or alimentary or colon or colorectal or "digestive system" or gastrointestinal* or gastro-intestinal* or "gi tract?")).mp.	766
50	operati*.mp. and (exp Abdomen/ or exp Digestive System Diseases/ or Digestive System/)	115981
51	(operati* and (abdomen or abdominal or alimentary or colon or colorectal or "digestive system" or gastrointestinal* or gastro-intestinal* or "gi tract?")).mp.	79469
52	pancreatectom*.mp.	15812
53	pancreaticoduodenectom*.mp.	9225
54	pancreatico-duodenectom*.mp.	204
55	(postoperat* and (abdomen or abdominal or alimentary or "digestive system" or gastrointestinal* or gastro-intestinal* or "gi tract?")).mp.	76207
56	(post-operat* and (abdomen or abdominal or alimentary or "digestive system" or gastrointestinal* or gastro-intestinal* or "gi tract?")).mp.	5974
57	(postprocedur* and (abdomen or abdominal or alimentary or "digestive system" or gastrointestinal* or gastro-intestinal* or "gi tract?")).mp.	602
58	(post-procedur* and (abdomen or abdominal or alimentary or "digestive system" or gastrointestinal* or gastro-intestinal* or "gi tract?")).mp.	458

59	(postsurg* and (abdomen or abdominal or alimentary or "digestive system" or gastrointestinal* or gastro-intestinal* or "gi tract?")).mp.	1328
60	(post-surg* and (abdomen or abdominal or alimentary or "digestive system" or gastrointestinal* or gastro-intestinal* or "gi tract?")).mp.	1003
61	proctocolectom*.mp.	4479
62	quadrantectom*.mp.	510
63	(reoperat* and (abdomen or abdominal or colon or colorectal or "digestive system" or gastrointestinal* or gastro-intestinal* or "gi tract?")).mp.	11578
64	(reresect* and (abdomen or abdominal or colon or colorectal or "digestive system" or gastrointestinal* or gastro-intestinal* or "gi tract?")).mp.	42
65	(resect* and (abdomen or abdominal or colon or colorectal or "digestive system" or gastrointestinal* or gastro-intestinal* or "gi tract?")).mp.	63810
66	segmentectom*.mp.	2872
67	splenectom*.mp.	28952
68	subsegmentectom*.mp.	191
69	sub-segmentectom*.mp.	8
70	(surgeon* and (abdomen or abdominal or alimentary or "digestive system" or gastrointestinal* or gastro-intestinal* or "gi tract?")).mp.	14207
71	(surger* and (abdomen or abdominal or alimentary or colon or colorectal or "digestive system" or gastrointestinal* or gastro-intestinal* or "gi tract?")).mp.	257333
72	(surgical* and (abdomen or abdominal or alimentary or colon or colorectal or "digestive system" or gastrointestinal* or gastro-intestinal* or "gi tract?")).mp.	145067
73	(tumorectom* and (abdomen or abdominal or alimentary or colon or colorectal or "digestive system" or gastrointestinal* or gastro-intestinal* or "gi tract?")).mp.	107
74	(unresect* and (abdomen or abdominal or alimentary or colon or colorectal or "digestive system" or gastrointestinal* or gastro-intestinal* or "gi tract?")).mp.	4275
75	exp Digestive System Surgical Procedures/	359524
76	General Surgery/	38483
77	exp Abdomen/su	22138
78	post*ectom*.mp.	12358
79	post*otom*.mp.	2595
80	or/15-79 [GI Surgeries & related terms]	1024106
81	14 and 80 [Colon/Colorectal Cancer + GI Surgeries]	95569
82	Neoadjuvant Therapy/	19724
83	Antineoplastic Combined Chemotherapy Protocols/	137043
84	exp Antineoplastic agents/	1084912
85	combined modality therapy/ or chemoradiotherapy/ or chemoradiotherapy, adjuvant/ or chemotherapy, adjuvant/ or radiotherapy, adjuvant/	225579
86	exp Organoplatinum Compounds/	22756
87	neoadjuvant*.mp.	31870
88	neo-adjuvant*.mp.	2208
89	"5-Fluorouracil"/	40954
90	"5-Fluorouracil".mp.	28435
91	"5-FU".mp.	17144

92	fluorouracil.mp.	49956
93	Fluoropyrimidines.mp.	1060
94	FOLFIRI.mp.	1115
95	FOLFOX.mp.	2376
96	exp Oxaliplatin/	6155
97	Oxaliplatin*.mp.	9516
98	exp Capecitabine/	4182
99	Capecitabine?.mp.	5794
100	CAPEOX.mp.	135
101	CAPOX.mp.	146
102	irinotecan??.mp.	9565
103	exp Leucovorin/	9852
104	(preoperative adj4 (chemo* or radio* or combined modality or therap*)).mp.	18199
105	NACRT.mp. [NeoAdjuvant ChemoRadioTherapy]	97
106	nCRT.mp.	340
107	(perioperative adj4 (chemo* or radio* or combined modality or therap*)).mp.	3711
108	or/82-107 [Neoadjuvant & related terms]	1295768
109	81 and 108 [Colon/Colorectal Cancer + GI Surgeries + Neoadjuvant]	20283
110	Clinical Trial, Phase III.pt.	16302
111	Clinical Trial, Phase III/	16302
112	Clinical Trial.pt.	520993
113	Clinical Trials, Phase III as Topic/	9187
114	Comparative Study.pt.	1854161
115	Comparative Study/	1854161
116	Controlled Clinical Trial.pt.	93553
117	Controlled Clinical Trial/	93553
118	Controlled Clinical Trials as Topic/	5491
119	Cross-Sectional Studies/	318160
120	Double-Blind Method/	156241
121	Equivalence Trial.pt.	503
122	Equivalence Trial/	503
123	Equivalence Trials as Topic/	282
124	Evaluation Studies.pt.	247851
125	exp Case-Control Studies/	1055845
126	exp Cohort Studies/	1956602
127	exp Randomized Controlled Trial/	500871
128	exp Randomized Controlled Trials as Topic/	133630
129	Longitudinal Studies/	131160
130	Meta-Analysis as Topic/	17614
131	Meta-Analysis/	110859

132	Multicenter Studies as Topic/	18159
133	Multicenter Study.pt.	266482
134	Multicenter Study/	266482
135	Placebos/	34742
136	Pragmatic Clinical Trial.pt.	1304
137	Pragmatic Clinical Trial/	1304
138	Pragmatic Clinical Trials as Topic/	401
139	Prospective Studies/	529025
140	Randomized Controlled Trial.pt.	500124
141	Retrospective Studies/	799765
142	Systematic Review/ [New MeSH 2019]	121052
143	Systematic Review.pt. [New PT 2019]	121052
144	Systematic Reviews as Topic/ [New MeSH 2019]	3059
145	Validation Studies/	97924
146	Validation Studies.pt.	97924
147	("phase 3" or "phase3" or "phase III").mp.	50124
148	((multicenter* or multicentre*) adj2 (trial? or study or studies)).mp.	299236
149	((noninferiority or non-inferiority) adj4 (trial? or study or studies)).mp.	3716
150	((single or double or triple or treble) adj3 (blind* or mask*)).mp.	217811
151	(case control* adj2 (study or studies)).mp.	300731
152	(comparative adj2 (trial? or study or studies)).mp.	1902317
153	(conceal* adj2 allocat*).mp.	2495
154	(controlled adj1 clinical adj2 (trial? or study or studies)).mp.	123747
155	(cross-sectional* adj2 (study or studies)).mp.	339011
156	(equivalen* adj4 (trial? or study or studies)).mp.	4397
157	(evaluation adj1 (study or studies)).mp.	375451
158	(longitudinal* adj2 (study or studies)).mp.	163005
159	(meta-anal* or metanal* or metaanal*).mp.	162584
160	(overview? adj4 (review or reviews)).mp.	13823
161	(pragmatic adj2 (trial? or study or studies)).mp.	3249
162	(prospective* adj2 (study or studies)).mp.	609694
163	(retrospective* adj2 (study or studies)).mp.	829772
164	(superiority adj4 (trial? or study or studies)).mp.	2736
165	(systematic adj4 (review or reviews or overview or overviews)).mp.	147754
166	(validation adj1 (study or studies)).mp.	109046
167	cohort*.mp.	569181
168	placebo*.mp.	205778
169	quasirandom*.mp.	104
170	random*.mp.	1169235
171	clinical Trial, Phase II/	32094

172	Clinical Trial, Phase II.pt.	32094
173	Clinical Trials, Phase II as Topic/	7609
174	("phase 2" or "phase2" or "phase II").mp.	78307
175	or/110-174 [Studies]	5614137
176	109 and 175 [Colon/Colorectal Cancer + GI Surgeries + Neoadjuvant + Studies]	10426
177	exp neoplasm grading/ or exp neoplasm staging/	182189
178	Neoplasm Recurrence, Local/	115030
179	Neoplasm Invasiveness/	79778
180	Neoplasm, Residual/	10239
181	("stage four" or "stage iv").mp.	18957
182	t4?.mp.	44936
183	(neoplas* adj2 stag???).mp.	170823
184	(neoplas* adj2 grad???).mp.	23455
185	(cancer* adj2 stag???).mp.	31551
186	(cancer* adj2 grad???).mp.	3813
187	(tumo?r* adj2 stag???).mp.	34318
188	(tumo?r* adj2 grad???).mp.	26465
189	(downgrad* or down-grad*).mp.	2568
190	(node stage? or node staging).mp.	1465
191	(nodal stage? or nodal staging).mp.	1644
192	(node grade? or node grading).mp.	15
193	(downstag* or down-stag*).mp.	3307
194	stage iv?.mp.	20149
195	stage iv.mp.	18726
196	(stage adj1 iv?).mp.	20630
197	locally advanced.mp.	23186
198	locally invasive.mp.	1503
199	(margin or margins).mp.	78530
200	(restage? or re-stage? or restaging or re-staging).mp.	2884
201	(upstag* or up-stag*).mp.	2291
202	(histologic grade? or histologic grading).mp.	5522
203	(pathologic stage? or pathologic staging).mp.	4017
204	(tumo?r* adj2 regress*).mp.	12890
205	(tumo?r* adj2 respond*).mp.	2075
206	(tumo?r* adj2 respons*).mp.	28177
207	or/177-206 [Neoplasm Grade / Stage]	552968
208	176 and 207 [Colon/Colorectal Cancer + GI Surgeries + Neoadjuvant + Grade/Stage]	6095
209	exp animals/ not (exp animals/ and exp humans/)	4672546
210	208 not 209	6065
211	limit 208 to humans	6057

212	210 or 211	6065
213	from 212 keep 1-3999	3999
214	remove duplicates from 213	3988
215	from 212 keep 4000-6065	2066
216	remove duplicates from 215	2066
217	214 or 216	6054

Table S2. Distribution of inclusion and exclusion criteria for study participant eligibility. *[Online only].*

**Study participants eligibility**	**No. (%)**
Inclusion criteria [a]	
Age ≥18	13 (65)
T2 colon cancer	1 (5)
T3 colon cancer	7 (35)
T4 colon cancer	20 (100)
Known colon cancer mutational status	1 (5)
Absence of specified co-morbidities	8 (40)
Tumor considered unresectable	3 (15)
Tolerance to chemoradiotherapy	1 (5)
Exclusion criteria [a]	
Age >75	4 (20)
Palliative-intent resection	3 (15)
Intraoperative chemo- or radio-therapy	1 (5)
History of malignant disease	5 (25)
Presence of specified co-morbidities	9 (45)
Complete pathologic response to neoadjuvant therapy	1 (5)
Complete colonic obstruction	2 (10)
Patients who suffered early mortality	1 (5)

Table S3. Summary of conclusions from included studies (n=20). *[Online only].*

**Study Characteristic**	
Reported study conclusions	**No. [ref]**
Neoadjuvant therapy is feasible (*e.g., toxicity, morbidity*)	12 [15,20,37,38,21–24,26,33,35,36]
Neoadjuvant therapy is efficacious	16 [15,20,32,33,35–38,21–28]
Neoadjuvant therapy can spare adjuvant therapy	1 [20]
Reported study limitations	
Small sample size	9 [21,26,27,32,33,35–38]
Retrospective design	11 [21,25,38,26–28,30,31,35–37]
Single-center data	6 [21,30,31,36–38]
Short follow-up	4 [22,32,33,35]
Reliance on accurate coding of database	4 [25,27–29]
Lack of outcomes in database	3 [25,27,29]
Heterogenous NAT regimens used	4 [26,33,36,37]
Heterogenous adjuvant therapy regimens used	1 [35]
Single-arm study	2 [20,37]
Reported recommended next steps	
Validation of outcomes in randomized and/or prospective trials	12 [15,20,35,37,22,25–29,33,34]
Longer follow-up	5 [15,22,24,33,37)

Larger sample size	5 [15,28,33,34,37]
